# Physical distancing and the perception of interpersonal distance in the COVID-19 crisis

**DOI:** 10.1038/s41598-021-90714-5

**Published:** 2021-06-01

**Authors:** Robin Welsch, Marlene Wessels, Christoph Bernhard, Sven Thönes, Christoph von Castell

**Affiliations:** 1 Human-Centered Ubiquitous Computing, Ludwig-Maximilians-Universität München, Munich, Germany; 2 Department of Psychology, Johannes Gutenberg-Universität Mainz, Mainz, Germany

**Keywords:** Human behaviour, Social behaviour

## Abstract

In the wake of the COVID-19 pandemic, it has been mandated to keep enlarged distances from others. We interviewed 136 German subjects over five weeks from the end of March to the end of April 2020 during the first wave of infections about their preferred interpersonal distance (IPD) before, during, and after the COVID-19 pandemic. In response to the pandemic, subjects adapted to distance requirements and preferred a larger IPD. This enlarged IPD was judged to partially persist after the pandemic crisis. People anticipated keeping more IPD to others even if there was no longer any risk of a SARS-CoV-2 infection. We also sampled two follow-up measurements, one in August, after the first wave of infections had been flattened, and one in October 2020, at the beginning of the second wave. Here, we observed that IPD varied with the incidence of SARS-CoV-2 within Germany. Overall, our data indicated that adaptation to distance requirements might happen asymmetrically. Preferred IPD rapidly adapted in response to distance requirements, but an enlargement of IPD may partially linger after the COVID-19 pandemic-crisis. We discuss our findings in light of proxemic theory and as an indicator for socio-cultural adaptation beyond the course of the pandemic.

## Introduction

The COVID-19 pandemic has led to the implementation of drastic and omnipresent prevention measures. It has undeniably posed enormous societal, financial, and scientific challenges. The virus is transmitted by aerosols and droplet particles, predominantly during face-to-face social interactions^1^. Mandatory measures to combat the spread of the virus include wearing a face mask and maintaining a minimum interpersonal distance (IPD) of at least 1.5 m to 2 m^2–4^. Note, however, that distance requirements vary substantially between countries (e.g., France: 1.0 m; South Africa: 1.5 m; the USA: 1.8 m; Japan: 2.0 m). This should reduce the reproduction rate of the pandemic respiratory virus by around 38 percent^5^ and has so far proven its effectiveness^4^. However, we usually prefer an IPD of around 1 m when interacting with unfamiliar people^6,7^. Hence, the demand for larger physical distances contradicts our usual proxemic behavior^8^.

Unusually large IPD typically produces heightened arousal^9^, distress^10^, and changes in social signaling within social interactions^11^. Such effects may appear as a short-term reaction when adhering to physical distance requirements. Welsch et al.^8^ have discussed a wide range of short-term effects in detail. However, prolonged effects of physical distancing have not yet been investigated. This study will focus on two questions: first, how does physical distancing requirements change IPD preferences over the ongoing pandemic and after the pandemic, i.e., when there is no longer a substantial risk of a SARS-CoV-2 infection, and restrictions are lifted again accordingly? Second, to what extent do people adapt to the enlarged distance requirements over time? To investigate this, we have gathered longitudinal data on IPD preferences during a nation-wide lockdown in Germany from March to May 2020 and supplemented this with two follow-up measurements (mid-August and end of October 2020) to observe the mid-term stability of these effects. We demonstrate how a novel social norm, i.e., physical distancing, manifests in IPD in response to the COVID-19 pandemic.

## Interpersonal distance

Based on ethnographic observations, Hall^12^ defined four distinct spaces based on their radius and acquaintanceship with the other person: intimate space for the partner or family (0 – 45 cm), personal space only to be entered by close friends (45 – 120 cm), social space for the interaction with strangers (120 – 365 cm), and public space (365 – 762 cm). In a more recent study, Hecht et al.^7^ could empirically refine the boundaries of personal space. They observed that it spans a circular-shaped boundary with a radius of about 1 m around the person when encountering strangers. Since the prevention measures in the COVID-19 pandemic aim at increasing IPD to all people outside one's household, be they strangers or close friends, they interfere with the usually preferred IPD.

The intrusion of personal space typically results in discomfort and arousal^13,14^, prompts an enlargement of IPD in order to reinstate homeostasis (see Cartaud et al.^15^,Coello and Cartaud^16^,but also see Bailenson et al.^17^), and sometimes results in people leaving the social interaction^18^. Moreover, IPD expectations can be violated by standing too far away in social interactions, which likewise produces discomfort^10^. Both processes can be explained by equilibrium theory. It suggests that approach and avoidance forces regulate IPD and that any deviation constitutes a violation of IPD^17^. For instance, approach forces elicited by physical attraction^6^ bring people closer together. In contrast, avoidance forces such as the perceived or estimated social threat^15,16,19^, or trauma^20^ promote enlargement of IPD. Does SARS-CoV-2 constitute a similar avoidance force and consequently increases preferred IPD?

Indeed, in a recent study, Cartaud et al.^21^ demonstrated that subjects accepted smaller IPD to virtual agents wearing face masks compared to virtual agents without face masks, which the authors attributed to a reduction in perceived risk of infection. Experimental studies in the domain of stigma research have observed that subjects enlarged IPD when encountering people with an infectious disease. Mooney et al.^22^ asked subjects to place chairs for an interview with either a person allegedly suffering from Acquired Immune Deficiency Syndrome (AIDS), a homosexual person, a cancer patient, or another student. The distance between the chairs was significantly enlarged when subjects expected to interview a person who has AIDS, even when subjects were aware that they could not be infected. Likewise, fear of contagion and stigma^23–25^ increased IPD. Can this bring persistent adjustments of IPD in response to the SARS-CoV-2 pandemic?

## Aims and hypotheses

**Figure 1:**
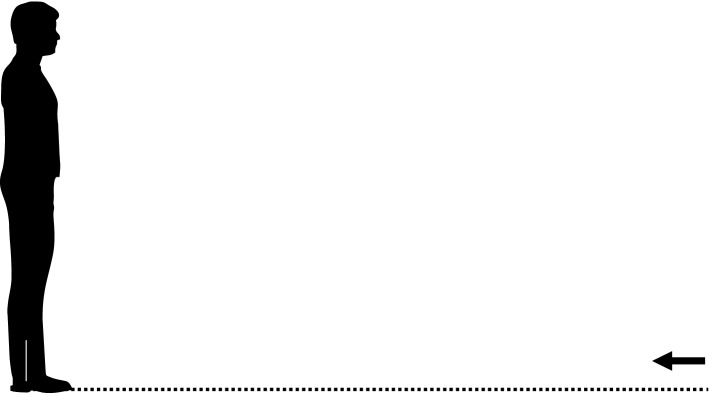
Male silhouette used in the present study. Subjects could place a point on the template to indicate preferred IPD.

The pandemic represents a unique case for proxemic research in which people reduce social contact and have to adhere to physical distance requirements. In a web-based task, we gathered IPD preferences over ten measurement points within five weeks for 136 subjects. More specifically, we assessed the currently preferred IPD during the pandemic (*peri-pandemic IPD*), the IPD retrospectively preferred before the pandemic (*pre-pandemic IPD*), and the IPD expected to be preferred after the pandemic (*post-pandemic IPD*) using an analog scale resembling the Interpersonal Visual Analog Scale^26^ and the Pedersen^27^ personal space scale, see Fig. 1. We will refer to this as *IPD measure* in the following.

This work investigated three different hypotheses. First, IPD adaptation processes have previously been observed on an individual level. For example, a personal crisis or traumatic event can strongly and persistently increase an individual's preferred IPD^20^. From cross-cultural studies on IPD, we also know that different social norms on IPD can coexist^28^. Therefore, we hypothesized (H1) that physical distance requirements change the social norm and thus increase IPD^8^ even after the COVID-19 pandemic. Specifically, we expected subjects to prefer a pre-pandemic IPD of around 100 cm, an enlarged peri-pandemic IPD consistent with the mandated physical distancing requirements of about 1.5–2 m, and a permanently enlarged IPD after the pandemic.

Second (H2), exposure to social isolation^29,30^ and loneliness^31^ have been shown to increase IPD preferences. Therefore, we suspected IPD preferences, especially peri-pandemic IPD, to slightly increase throughout the lockdown due to reduced social contact^8^.

Lastly, we conducted two follow-up measurements in mid-August and end of September 2020 to explore whether the effects persisted after the nation-wide restrictions were lifted and whether IPD relates to COVID-19 incidence.

## Results

### Time and distance

To compare the three IPD measures (H1) and to evaluate the effect of the duration of physical distancing requirements on IPD preferences (H1 and H2), we constructed a Bayesian linear mixed-effects model. Following a Bayesian approach, we relied on the posterior *p*-value (*p*_*b̃*_*),* and the 95% high-density posterior intervals (HDI) supplemented with the mean of the posterior for statistical inference*.* The model's population-effects term included the IPD measure (pre-pandemic IPD as baseline vs. post-pandemic IPD and peri-pandemic IPD) and the time passed since the first measurement in the first survey period (1, 5, 8, 12, 15, 19, 22, 26, 29, 32 days). Because we were interested in the change of IPD measures over time (H2), we added the interaction effect between IPD measure and time passed to the population-effects term. The IPD judgments represented the criterion in all analyses. To account for the data's repeated measures structure and the potential effect of silhouette gender, we added a subject-specific intercept term and a varying slope for the silhouette's gender (male vs. female).

In total, this model explained *R*^2^ = 77.17% [75.87; 76.80] of the variance in the IPD data; intercept = 121.53 [115.02 ;127.95]. There was a strong main effect of IPD measure. Both peri-pandemic IPD, *b* = 60.68 [57.92; 63.40 ]_,_ *p*_*b̃*_ = 0.00%, $\updelta$_*t*_ = −1.38 [1.25; 1.52] and post-pandemic IPD differed from pre-pandemic IPD, *b* = 19.61 [16.87;22.32]_,_ *p*_*b̃*_ = 0.00%*, *$\updelta$_*t*_ = 0.45 [0.37; 0.52]. Consistent with our hypotheses, subjects reported that they preferred a mean distance of 118 cm (*SD* = 37 cm) before the pandemic, which fitted the preferred IPD of around 100 cm to 115 cm observed in previous studies^6,7,10^. Within the pandemic, subjects largely adopted to the physical distancing requirements and, on average, preferred a mean peri-pandemic IPD of 183 cm (*SD* = 43 cm). Subjects also anticipated this increased distance preferences to partially persist after the pandemic, with an average post-pandemic IPD of 141 cm (*SD* = 44 cm).

**Figure 2:**
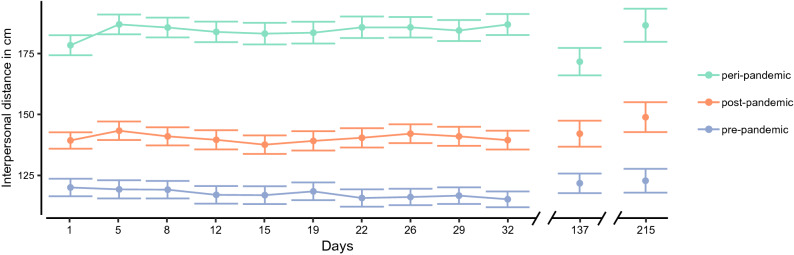
Interpersonal distance in cm as a function of days since the first measurement (one week after the beginning of the first lockdown in Germany) and IPD measure (peri-, post-, pre-pandemic). The IPD measures were averaged across the silhouette gender.

To investigate the effect of reduced social contact (H2) and the course of adaption (H1), we will now closely look on the model parameters quantifying the effect of time. Pre-pandemic IPD decreased slightly over time by about 9 cm, *b* = −0.18[−0.33; −0.03]_,_ *p*_*b̃*_ = 0.98%*, *$\updelta$_*t*_ = 0.00 [−0.01; 0.00]. Consistent with previous correlational findings on reduced social contact^29,31^, we could show that the change in IPD over time was larger for the peri-pandemic IPD than for the pre-pandemic IPD, *b* = 0.32[0.18; 0.46]_,_ *p*_*b̃*_ = 0.00%*, *$\updelta$_*t*_ = 0.01[0.00; 0.01]. Peri-pandemic IPD increased by about 8 cm over the course of our study period, descriptively. There was also a meaningful change in post-pandemic IPD over time when contrasted to the trajectory of pre-pandemic IPD, *b* = 0.17 [0.03; 0.31]_,_ *p*_*b̃*_ = 0.94%*, *$\updelta$_*t*_ = 0.00 [0.00; 0.01]. It increased about 3 cm over the first ten measurement points. In addition, there was a small but distinguishable variation with regard to silhouette gender, varying-slopes of silhouette gender (factor coded as male = −0.5 vs. female = 0.5) with *SD* = 0.90 [0.00; 1.80]. In line with previous research^6,7,32^, preferred IPD for male silhouettes was slightly enlarged (*M* = 147 cm; *SD* = 37 cm) compared to female silhouettes (*M* = 149 cm; *SD* = 37 cm). This gender effect was largely stable across the measurement period (for a figure resembling Fig. 2 split for gender see Supplementary Fig. S1 online) and thus validates our measurement and the inclusion of silhouette gender as a varying-slope in the statistical model.

### Controlling for physical distancing

In addition to the mere graphical judgment of IPD, we asked subjects to indicate their physical distancing norm. Averaged across the first 10 measurements, subjects deemed a physical distancing norm of 172 cm (*SD* = 29 cm) appropriate during the pandemic. None of the subjects reported a norm smaller than 150 cm throughout our study, which indicates that all subjects were consistently aware of the physical distancing requirements posed by the COVID-19 pandemic. This explicit statement on distance requirements allowed us to demonstrate the validity of our results in two respects. First, if our measurement is valid, then IPD norms and IPD preferences should roughly coincide (H1). Indeed, when calculating the mean difference between the peri-pandemic IPD and the reported physical distancing norm at every measurement point, peri-pandemic IPD exceeded the social norm only by about 6 cm on average (*SD* = 51 cm), which supports H1. Second, when looking closely at Fig. 2, one can see a slight increase in peri-pandemic IPD accompanied by a slight decrease in pre-pandemic IPD. We constructed an additional linear mixed effect model to control for this potential confound of contrasting across time. In this model, we compared the physical distancing requirements to peri-pandemic IPD.

**Figure 3:**
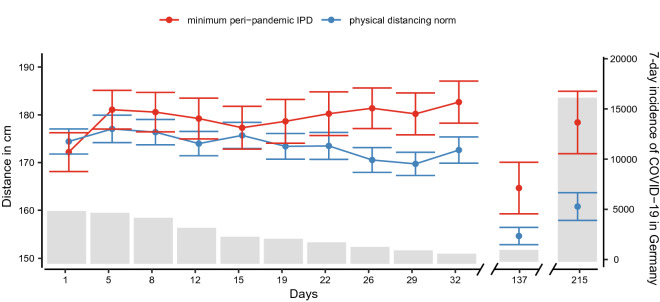
Physical distance norm and minimum interpersonal distance (IPD in cm) during the pandemic averaged across the silhouette gender (left y-axis), and 7-day incidence of COVID-19 cases (right y-axis) as a function of days since the first measurement (one week after the beginning of the first lockdown in Germany).

We selected the minimum individually preferred peri-pandemic IPD (to male or female silhouette) at a given measurement and subtracted the physical distancing norm reported at the individual measurement dates (day 1 until day 32) from it. This bias score, indicating the difference between physical distancing norm and peri-pandemic IPD, was then again modeled as a function of time, adding subjects as varying intercept term. This permitted the validation of the effect of time on peri-pandemic IPD, see Fig. 3. While at the beginning there was nearly no difference between norms and minimum peri-pandemic IPD (*Mdiff* = −2 cm; *SDdiff* = 50 cm), the difference increases to about 10 cm (*SD* = 52 cm), Fig. 3. This bias can be distinguished from pure noise within our model, *b* = 0.38[0.17; 0.59]_,_ *p*_*b̃*_ = 0.04%*,* $\updelta$_*t*_ =0.01 [0.00; 0.01]. Therefore, when controlling for the effect of physical distancing norms on IPD preferences, peri-pandemic IPD still increased as a function of time, lending support to H2.

Most alarmingly, the reported physical distancing norm tended to decrease over time (*r* = −0.74), in accordance with the incidence, *r* = 0.87. We calculated a point estimate as any inference is biased by the follow-up and the trend can be verified by inspecting Fig. 3. Thus, both the time since physical distance requirements were implemented and the incidence of COVID-19 seem to affect the individual physical distance norm.

### Exploring IPD at follow-up

So far, we have analyzed the measurements from day 1 to day 32. That is the effect of the pandemic on IPD over five weeks during and after the first lockdown. We were also interested in the pandemic's progress and its impact on IPD and thus secured two additional measurements. We now explore the two follow-up measurements in mid-august (day 137), when the incidence of COVID-19 in Germany was relatively low, and end of September, amid the second wave of COVID-19 cases (day 215), relative to day 32, which marks our last measurement within the first wave of infections. Descriptively, peri-pandemic IPD dropped on day 137, see Figs. 2 and 3, but was reinstated on day 215. We can also see a substantial increase in post-pandemic IPD on day 215, accompanied by a small rise in pre-pandemic IPD.

**Table 1:** Parameters of the Bayesian *t*-tests for the comparisons between the two follow-up dates and day 32, separate for each IPD measure; day 137 with *N* = 66 and day 215 with *N* = 52.

Comparison		*b* [95% HDI]	$\updelta$_*t*_ [95%HDI]	*p*_*b̃*_
**Pre-pandemic**				
32 vs. 137		4.91 [0.60; 9.16]	0.18 [0.02; 0.34]	1.29%
32 vs. 215		4.34 [−1.13; 9.87]	0.13 [−0.04; 0.29]	6.14%
**Peri-pandemic**				
32 vs. 137		−16.58 [−22.75; −10.31]	−0.37 [−0.52; −0.23]	0.00%
32 vs. 215		−2.70 [−8.11; 2.59]	−0.05 [−0.16; 0.06]	16.10%
**Post-pandemic**				
32 vs. 137		2.21 [−2.19; 6.67]	0.06 [−0.06; 0.17]	16.09%
32 vs. 215		8.57 [4.14; 13.15]	0.21 [0.09; 0.32]	0.01%

To establish whether these trends can be substantiated, we separately tested the individual IPD differences between day 32 and day 137 as well as between day 32 and day 215 (*N* = 66 for day 137 and *N* = 52 at day 215) against 0 (effect coding with −0.5, + 0.5). Again, we added subject-specific intercepts and varying slopes for the silhouettes to the model. Additionally, we fitted the residuals to a studentized distribution to control for potential outliers in this subset of our data and allow for better shrinkage, resembling a Bayesian *t*-test. Compared to day 32, we found a decrease in peri-pandemic IPD on day 137, but not on day 215. This increase in IPD contradicts the previous trend in the data. Also, in contrast to the previous trend, we found a retrospective distortion of reporting relatively larger pre-pandemic IPD, which was statistically distinguishable for day 137, see Table 1.

For post-pandemic IPD, the effect was distinguishable on day 215 (see Table 1), where post-pandemic IPD was around 6 cm larger than on day 32. That is, at the end of our study and at the wake of the second wave, subjects predicted post-pandemic IPD to nearly coincide with the minimum physical distance (150 cm) requirement demanded during the pandemic.

## Discussion

Physical distancing requirements pose a challenge to our proxemic behavior. Our study demonstrates that enlarged physical distancing requirements were adopted in our sample of the German general public. Subjects preferred a considerably increased peri-pandemic IPD compared to the distance they chose before the pandemic. Interestingly, these enlarged IPD preferences were judged to persist to some degree post-pandemic. On average, our subjects anticipated a post-pandemic IPD of 140 cm.

Furthermore, peri-pandemic IPD preferences slightly increased at the beginning of our survey, even when controlling for knowledge about the IPD norms. Therefore, we can speak of a genuine preference for larger IPD during the pandemic beyond the mere adoption of physical distancing requirements. Also, we found evidence for an increased pre-pandemic IPD at our follow-up. At the end of 2020, subjects retrospectively reported that they had kept larger distances before the pandemic. Note that this effect of a retrospective distortion is novel in the field of proxemic research and needs to be investigated closer in the future. Still, it validates our choice of comparing pre-, peri-, and post-pandemic IPD at every measurement. Our study can inform proxemic theory as it showcases that IPD can adapt to physical distancing requirements. In the face of external hazards, such as the risk of a SARS-CoV-2 infection, people rapidly adapt to a new social norm for IPD^33–35^. Although stable social norms have been described since the beginning of proxemic research—e.g., Japanese who, on average, prefer a larger IPD than Germans^12,28,34^—a dynamic process of adaptation^8^ has not been observed empirically so far.

Furthermore, we observed a drop of about 20 cm in peri-pandemic IPD despite continued physical distancing requirements in our follow-up period. This drop may be related to a pronounced decrease in the COVID-19 incidence rate in Germany in mid-August. Thus, one could hypothesize that IPD preferences are not only associated with the mandated distance requirements. They might also be closely calibrated to the perceived individual risk of infection and, thus, the individually perceived importance of physical distancing. This is in line with proxemic theories emphasizing IPD as a means to ensure safety from others^36,37^ as well as with a recent study indicating reduced IPD in response to mask use^21^.

Notably, post-pandemic IPD was significantly enlarged at the end of September (day 215), such that it coincided closely with the minimum physical distance requirements of 1.5 m issued during the pandemic in Germany. This suggests that distance requirements can be quickly adapted, but also that enlarged IPD preferences may partially linger after restrictions are lifted, and the risk of infection with SARS-CoV-2 is unlikely. Thus, the IPD social norm could be enlarged in the long term. However, future research has to underpin our findings when the pandemic is over.

From a proxemic perspective, it seems plausible that IPD may adapt asymmetrically over time. The asymmetrical adaption describes a rapid increase of the preferred IPD in the scope of the pandemic, while the preferred IPD after the pandemic decreases rather slow. This is in line with our previous work^10^. Discomfort due to extrusion, e.g., 10 cm away from personal space boundaries, is typically smaller than an intrusion, e.g., intruding 10 cm into personal space. Having said this, there is an obvious need for future research to empirically test these considerations. Longitudinal behavioral data must be obtained on the maintenance of post-pandemic IPD to investigate the hypothesis of an adaptive asymmetry.

In line with this hypothesis, we found that a reduction in social contact can be related to a slow but steady enlargement of IPD. Previous studies^29–31^ have either related loneliness in a cross-sectional design or manipulated social isolation by keeping people alone in a room over hours. Here, we can present the first empirical evidence for a longitudinal relation of reduced social contact and IPD. Therefore, both rapid but also slow adaptation processes appear to interplay here.

Our study can also inform the measurement of proxemic patterns. Hayduk^13^ described that real-world and survey-based judgements of IPD could diverge significantly. We only found a slight bias towards an overestimation of IPD as compared to distancing requirements. Also, in line with this critique, the effect sizes observed in our study were comparably small. Nevertheless, effects appeared persistently across time, see Fig. 2. We therefore suggest that the employed measure of IPD was practically useful as it could be deployed online, i.e., not harming subjects by acquiring behavioral data, and without high-quality visualization, e.g., using 3D software with a virtual character wearing masks^21^. Still, our measure could be biased as it did not measure actual spatial behavior. It considered retrospective or anticipative judgments that may not exactly align with observations of natural behavior or behavioral tasks^13^.

IPD may operationalize the adaptation and manifestation processes of social norms that occur on a socio-cultural level in response to a pandemic. For this, it was essential to investigate IPD in a longitudinal design. Our study can lend credibility to a more representative but cross-sectional study of Cartaud et al.^21^, who showed that local variation of SARS-2-CoV incidence relates to IPD on a cross-sectional level. Here, we could establish a temporal relationship across the follow-up measurements within-subjects at different stages of the pandemic. With this in mind, the SARS-CoV-2 pandemic could be considered a natural experiment in which one can witness the adaptation and manifestation of social norms.

Having said this, a few caveats should be considered. First, we have sampled IPD within subjects, which inevitably induced a correlation between measures. Therefore, local variation or calibration to risk could have been underestimated in our study as subjects were giving relatively consistent distance estimates across measurement points. Second, our study sample was non-representative of the German population, since two-thirds of our subjects were university students, and the mean age of the sample was around 30. Therefore, one could argue that, on average, our sample might have been less exposed to inevitable social contacts and thus at a lower risk of infection compared to essential workers^38^, and was at a lower risk of severe symptomatology or a fatal outcome in case of a SARS-2-CoV infection compared to old-aged adults^39^. Therefore, adjustments due to perceived risk might have been underestimated in our study. In line with these considerations, effects over time on IPD measures were relatively small. Future studies should investigate more closely how changes in preferred IPD over time can be optimally measured. Taken together, the limitations discussed may have led to an overly conservative estimate of the reported effects, which, however, should not contradict the overall validity of our findings.

We conclude that IPD preferences are highly flexible, adapt quickly to situational demands, and, thus, vary across the pandemic. Furthermore, the adaptation to IPD norms may happen asymmetrically; subjects adapt to physical distance requirements quite fast by keeping larger IPD but report to prefer an enlarged post-pandemic IPD. At the end of our study and at the wake of the second COVID-19 wave, subjects estimated their post-pandemic IPD to be similar to the physical distancing requirement during the pandemic of about 150 cm.

## Method

### Subjects

As preregistered (As Predicted #38,026), we set the required sample size to a minimum of 50 subjects. This sample size was determined based on data simulation considering an optimal level of statistical power (e.g., a detectability of true effects above 80% with an α-level of 5%). The study was approved by the local ethics committee of the University of Mainz (2020-JGU-psychEK-S003).

Subjects were recruited via online advertisements of the University of Mainz and the LMU Munich and associated online communities. In total, 186 subjects subscribed to the mailing list to voluntarily participate in the survey. Subjects responding to the demographic questions of the first measurement point and completing at least 7 of the ten measurement points (70%) of the survey were included in the analyses. The resulting sample comprised 136 adults at day 1 (114 female, age: *M* = 29.40 years, *SD* = 11.75 years) with a minimum of *N* = 122 at day 12. All subjects gave written informed consent at the beginning of each measurement point, in accordance with the Declaration of Helsinki, and declared to be resident in Germany. The latter ensured similar pandemic-related external factors for all subjects. 87 of the 136 subjects were in a relationship. About two-thirds (94 of 136 subjects) were enrolled students. Two subjects became infected with COVID-19 during our study. Subjects did not receive any monetary compensation but could obtain partial course credit for participation in the study.

We partly deviate from the preregistered analysis plan so far as we have added more measures to the research design to suit the pandemic's unexpected course. We also encountered a much larger sample than expected, which allowed more careful modeling of our data. The preregistered analysis is available here: https://osf.io/p3ek4/?view_only=9c46dba62e124c0b88a704a2c9e177d0.

### Survey

The survey was conducted from 30 March to 1 May 2020 and comprised ten measurement points (2 per week). Two additional measurement points followed this preregistered first survey period in mid-August (*n* = 66) and the end of October 2020 (*n* = 52) to observe longitudinal effects in the unexpectedly long-lasting pandemic. At the first measurement point, participants completed a battery of demographic questions before they proceeded to the survey's main part. The latter was identical for all measurement points, included 14 items, and took approximately 5–10 min to process. The 14 items covered questions regarding the participants' time perception, emotional state, and IPD preferences.

This article focuses on the 6 IPD items resembling the Interpersonal Visual Analog Scale^26^ and the Pedersen^27^ personal space scale, see Fig. 1. These items instructed the participants to place a point at their preferred distance from a depicted silhouette using a mouse click.

We included two items for each of the three IPD measures, one displaying a female and the other a male silhouette (see Fig. 1). Subjects were instructed as follows: "Imagine you are in a strange city and want to ask someone for directions. You see this strange men/women and you want to approach him/her. How much distance did/do/will you keep from a strange person in public before/within/after the Corona crisis?" [German original version: "Stellen Sie sich vor, Sie wären in einer fremden Stadt und möchten jemanden nach dem Weg fragen. Sie sehen diese/n fremde/n Frau/Mann und möchten ihn/sie ansprechen. Wieviel Abstand hielten/ Sie vor/während/nach der Corona-Krise zu einer fremden Person in der Öffentlichkeit?"]. Note that this scenario and instruction have proven to be both reliable and valid^6,7,10,34,40–42^.

The items were presented in a fixed order (pre-pandemic, peri-pandemic, post-pandemic; first showing the female and then the male silhouette for each measure). Both silhouettes represented upright standing adults with a height of 170 cm, which is nearly the mean height of the German population^43^. The actual pixel height of the presented silhouettes was 368 pixels. The dotted line, which represents the range for the subjects' indication of their preferred IPD, was about 1.59 times longer than the silhouette's height. Subjects could state IPD preferences from 0 to a maximum of 270 cm, which is well beyond the mandated physical distancing requirements and exceeds typical and even extreme IPD preferences. To gather the IPD measure, we recorded the horizontal distance in pixels between the placed point and the silhouette and converted it into cm relative to the silhouette size. For this, we divided the distance in pixels by the silhouette's height in pixels and multiplied it with the represented silhouette's height of 170 cm. The resulting formula was: $IPD\;in\;cm = \frac{{horizontal\;distance\;in\;pixel}}{{silhouette\;height\;in\;pixels}}*170\;cm$. The last IPD item captured the participants' factual knowledge about the currently mandated physical distancing norm ("How much distance should currently be kept when addressing a stranger in public?"). Responses were given as a number integer entry in cm.

### COVID-19 pandemic in Germany during the survey period

The survey started one week after the beginning of a first lockdown in Germany (30 March 2020; incidence: 4124; 7-day incidence: 5259, Robert Koch Institute, RKI). At that time, an IPD of 150 cm to 200 cm was mandated. People were only allowed to meet one other person in public. Schools, restaurants, and shops were closed. The first lockdown reduced the COVID-19 incidences (see Fig. 3) and was gradually lifted until mid-May 2020. For example, schools opened again on 4 May 2020, which corresponds to day 35 of our survey period (incidence: 724; 7-day incidence: 884 RKI). One week later, restaurants were again allowed to receive guests under strict hygiene regulations. However, physical distancing regulations persisted, and the obligation to wear face masks in shops and public transport was additionally introduced. The first follow-up measurement was conducted on 14 August 2020 during the summer holidays when incidences were still low (incidence: 1374; 7-day incidence: 1200, RKI). In addition to physical distancing and hygiene rules, travelers returning from risk areas had to present a negative SARS-CoV2 test or stay in a two-week quarantine. In contrast, the period around the second follow-up was characterized by exponentially growing incidences (incidence: 19,424; 7-day incidence: 16,340, RKI). Therefore, a second but lighter lockdown was officially announced (day 213, 28 October 2020) to start in the following week.

### Statistical analyses

We excluded outliers in our IPD measures using the Tukey criterion: trials three times the interquartile-range lower than the first or higher than the third quartile of the IPD judgments across all measurement points were discarded separately for each subject and IPD measure (pre-pandemic IPD, peri-pandemic IPD, post-pandemic IPD). We also excluded missing values. In total, we excluded 2.15% (182 of 8346) data points.

We used brms^44^, a wrapper for the STAN-sampler^45^ for R to model our data. We applied normally-distributed priors (*M* = 0, *SD* = 1) on all population-level effects, with Cholesky priors on the unstructured (residual) correlation (η = 2), and a *t*-distributed prior (*df* = 3, *M* = 0, *SD* = 1) on the intercept. The variance parameters were scaled to the respective mean and standard deviation of the target parameter. We computed 4 Hamilton-Monte-Carlo chains with 20,000 iterations each and 10% warm-up samples. Trace plots of the Markov-chain Monte-Carlo permutations were inspected for divergent transitions. All Rubin-Gelman statistics^46,47^ were well below 1.1. We explored the effect of different weakly informative priors on the data. None had an effect on statistical inference.

*p*_*b̃*_﻿ was computed by calculating the relative proportion of posterior samples being zero or opposite to the median^46^ and should have properties similar to the classical *p*-value^48^. Effects were considered meaningful when there was a particularly low probability (*p*_*b̃*_﻿ ≤ 2.50%) for the effect being zero or opposite. To provide a standardized estimate of the effects, we calculated $\updelta$_*t*_, which can be interpreted similarly to Cohen's *d*^49^. The data of this study is available here: https://osf.io/w9ej7/?view_only=52ee9b86507a4faeb74c9dba02466a60.

## Supplementary Information

Supplementary Information.
