# Supplementary material for: Physical distancing and the perception of interpersonal distance in the COVID-19 crisis
Source: Sci Rep. 2021 Jun 1;11:11485. doi: 10.1038/s41598-021-90714-5 (PMC8169674; doi:10.1038/s41598-021-90714-5)
Supplement: Supplementary file 1 — Supplementary Information. [file 41598_2021_90714_MOESM1_ESM.pdf]

Supplementary Information to

Physical distancing and the perception of interpersonal distance in the COVID-19 crisis

Robin Welsch[1], Marlene Wessels[2], Christoph Bernhard[2], Sven Thönes[2] & Christoph von Castell[2]

[1]Human-Centered Ubiquitous Computing, Ludwig Maximilian University of Munich, Germany

[2]Department of Psychology, Johannes Gutenberg-Universität Mainz, Germany

Supplementary Figures

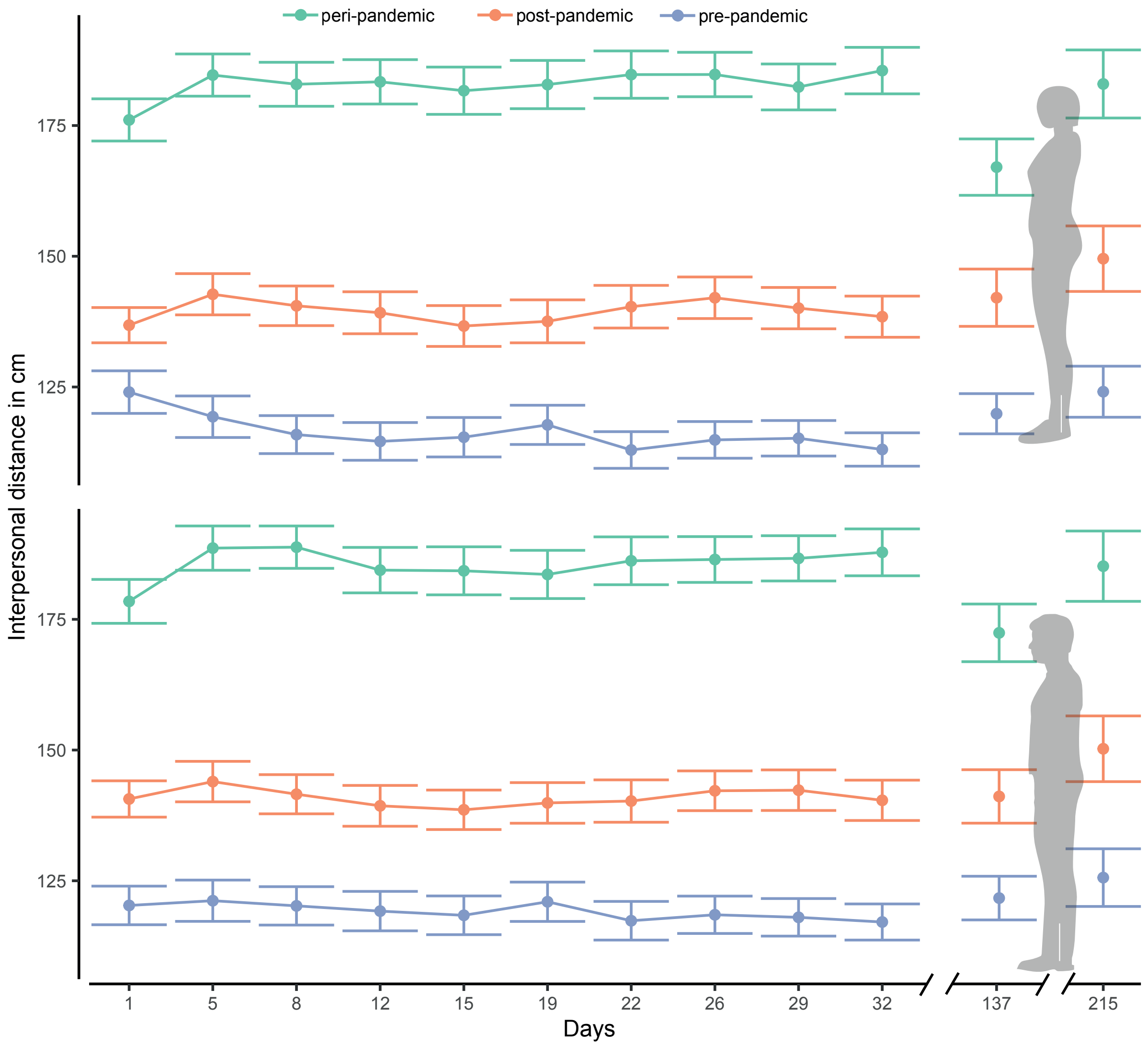

Figure S1. Interpersonal distance in cm as a function of silhouette gender (female at top; male at botom) and days since the first measurement (one week after the beginning of the first lockdown in Germany) and IPD measure.
